# Emotional impact and psychological aspects involving treatments with donated oocytes

**DOI:** 10.5935/1518-0557.20250008

**Published:** 2025

**Authors:** Vanessa Devens Trindade, Débora Farinati, Natália Fontoura de Vasconcelos, Victória Campos Dornelles, Isadora Badalotti-Teloken, Sophia Abur, Talita Colombo, Alvaro Petracco, Marta Ribeiro Hentschke, Carlos Eduardo Poli de Figueiredo, Mariangela Badalotti

**Affiliations:** 1 Fertilitat - Reproductive Medicine Center; Porto Alegre, RS, Brazil; 2 Pontifical Catholic University of Rio Grande do Sul, PUCRS. Graduate Program of Medicine and Health Sciences. Porto Alegre, Rio Grande do Sul, Brazil

**Keywords:** oocyte donation, reproductive techniques, infertility, psychological aspects

## Abstract

**Objective:**

The number of cycles performed with egg donation increases every year. In Brazil, between 2020 and 2023, 3.754 pregnancies were obtained with the aid of egg reception. The main objective of this study was to evaluate the emotional impact and psychological aspects involving treatments with heterologous oocytes.

**Methods:**

Data was collected between January 2021 and December 2022. In total 39 receptors answered the questionnaire.

**Results:**

All of them had partners with an average of 10.6 years of relationship (SD±5.7) and a mean of 5.3 years trying to conceive (SD±3.0). For 55.2% egg donation was a good treatment option, 31.5% thought it was a difficult decision at first but accepted it well later and 13.1% only accepted because they had no other option. Regarding reported emotions, 58.9% of participants felt calm even though 64.1% also felt anxious. In addition, 62.1% considered it important for their child’s pediatrician or doctor to know that conception took place through egg donation and 67.5% agreed at least in part that it was important to tell their child how they conceived.

**Conclusions:**

The study showed that most patients accepted egg donation as a good treatment option. Feelings such as anxiety and tranquility were presented in most patients, reflecting the variety of emotions concurrent with this process. The dilemma of whether to tell their offspring about their genetic origin was presented, in agreement with previous studies.

## INTRODUCTION

Over the last few decades, we have witnessed a revolution in the fields of technology, culture and human relations, and the contemporary family is a reflection of these changes. Today’s family is different from what is known as the nuclear family, made up of father, mother and children. New family structures are gaining ground and changing the landscape of family relationships, often with kinship ties that were once non-existent (Farinati & Lopes, 2015). Every year, the number of families built with the help of reproductive medicine grows significantly. New technologies not only facilitate the formation of traditional families, but also make more modern families possible, such as those created through the participation of a third party in conception, including families formed from donated gametes (Blakemore *et al*., 2019; Ghelich-Khani *et al*., 2020; Imrie *et al*., 2022; Roudinesco, 2003). This advance in human reproduction has made it possible for women with ovarian factor infertility to also experience the possibility of pregnancy.

The first successful case of egg donation was reported in 1984 (Lutjen *et al*., 1984). This technique was initially intended for women with premature ovarian insufficiency (POI), which is defined as a temporary or definitive failure of gonadal function that occurs after menarche and before the age of 40. Nowadays, egg donation has had its indications expanded and is constantly evolving (Colombo *et al*., 2023).

A woman has a finite number of oocytes. During the stages of fetal development, all the oocytes a woman will have throughout her life are produced. Over time there is a natural decline in the ovarian reserve until the menopause, with a reduction in the quality of the oocytes (Baird *et al*., 2005). When associated with other risk factors, such as smoking, radiation, chemotherapy or autoimmune disease, this loss can be accelerated (de Vet *et al*., 2002; Legendre *et al*., 2014). The decrease in ovarian reserve comes to light with the increasingly late search for pregnancy, driven by personal projects - such as career advancement and financial goals - or social aspects, such as the absence of a partner (Braga *et al*., 2016).

Fortunately, patients with infertility secondary to follicular depletion can undergo assisted reproduction treatments through egg donation. The search for egg donation in general comes after unsuccessful attempts to conceive with one’s own oocytes. It is essential to note that the couple who come in for egg donation in most cases have already traveled a long way in their search for a biological child. For them a long time has passed since the initial diagnosis of infertility, the investigation into its causes and the most varied therapies. This whole process is marked by hopes and frustrations and causes wounds of intense and varied depths in the uniqueness of each reproductive story (Lasheras *et al*., 2020).

Given the advances in reproductive medicine and the increasingly late age at which people become pregnant, the number of oocyte donation cycles is increasing every year (Greenfeld, 2015). Data from the Embryo Production Report (SisEmbrio) reveals that, in Brazil, from 2020 to 2023, 3.754 clinical pregnancies were obtained using donated oocytes (fresh oocytes 923, frozen oocytes 2.831) (ANVISA, 2024). It is essential to address the emotional aspects of egg donation treatment. The complexity of this scenario involves issues such as giving up one’s genetic load and whether or not to tell a child his or her biological origin. Thus, the objective of the present study was to evaluate the emotional impact and psychological aspects involved in the treatment with donor oocytes.

## MATERIAL AND METHODS

A Cross-sectional observational study was carried out at an Assisted Reproduction Center in Porto Alegre, Brazil. The study included women submitted to *in vitro* fertilization (IVF) using donated oocytes from an international egg bank or an egg-sharing program.

Patients who were candidates for egg reception were invited to answer an electronic questionnaire, developed on the web-based platform (Qualtrics XM) - by a multidisciplinary team made up of specialists in assisted reproduction and mental health. All patients who agreed to take part in the study and complete the online questionnaire were included and signed the Informed Consent Form. A total of 50 patients were invited to enter the study, 40 agreed to take part and 39 completed the questionnaire.

Data was collected between January 2021 and December 2022. Continuous variables were presented as mean±standard deviation or median and interquartile range, as appropriate. Categorical variables were presented as frequency and percentage. This study was approved by the Research Ethics Committee of Pontifical Catholic University of Rio Grande do Sul (CAAE 50170521.4.0000.5336). All authors signed a confidentiality responsibility term before collecting data and all patients signed an informed consent.

To examine potential differences in emotional responses among women who received donated eggs, statistical comparisons were conducted between two main groups based on their levels of agreement or disagreement with three specific statements. Data were analyzed using *Statistical Package for the Social Sciences* (SPSS), with Fisher’s exact test for 2x2 contingency tables, which is suitable for small sample sizes and binary variables. For contingency tables with more than two categories, the chi-square test was applied. All analyses aimed to determine whether statistically significant differences existed in emotional responses, such as feeling “calm,” “fearful,” or “optimistic,” in relation to each of the three statements analyzed, with a *p*-value threshold of 0.05 set for statistical significance.

## RESULTS

From 39 egg recipients that answered the questionnaire, all of them had partners, with an average time of relationship of 10.6 years (SD ±5.7). A total of 12.5% women reported having children with their current partners and 20% children from a previous relationship. Also, 12.8% of the patients’ partners had children from previous relationships. Regarding time spent trying to conceive, the average was 5.3 years (SD±3.0). Most patients (84.6%) reported having someone with whom they could share their feelings. Regarding the patient’s acceptance of receiving donated eggs, 55.2% believed it was a good option, 31.5% thought it was a difficult decision at first but accepted it well later and 13.1% accepted because they had no other option.

The results regarding the feelings mentioned by the patients are shown in Table 1. They were asked to answer ‘*yes*’ or *no’* to all the feelings listed. Table 2 shows the responses regarding psychological or psychiatric treatment.

**Table 1:** Feelings expressed by egg recipients.

Feeling, number (%)	Yes
Calm	23 (58.9)
Anxious	25 (64.1)
Frightened	17 (43.5)
Optimistic	35 (89.7)
Hopeful	38 (97.4)
Anguished	9 (23.0)

Number of patients = 39.

**Table 2:** Psychological or psychiatric treatment in egg receiver patients.

Question, number (%)	Yes
Have you ever received any psychological or psychiatric treatment?	23(58.9)
Are you currently in psychological or psychiatric treatment?	11(28.2)
Are you using any medication for depression/anxiety/insomnia?	10(25.6)

Number of patients = 39.

The last section of the questionnaire asked patients to select how they felt about aspects related to parenting through egg reception. The results are shown in Table 3. In total, 37 patients answered these questions.

**Table 3:** Aspects related to parenting through egg reception.

Question	Agree n (%)	Partially agree n (%)	Disagree n (%)
Kids born from donated eggs are the same as any other children	36 (97.3)	1 (2.7)	(0.0)
My genetic connection with the child will be the same as my partner’s	22 (59.4)	9 (24.3)	6 (16.2)
It does not matter how much a woman loves her child born through egg donation, she will never feel like it is really hers	2 (5.4)	(0.00)	35 (94.5)
It is hard to accept the loss of my genetic load	5 (13.5)	18 (48.6)	14 (37.8)
The feelings that surround having a child conceived through gamete donation bothers me	2 (5.4)	7 (18.9)	28 (75.6)
You fear that the donor will regret her decision and will start looking for the child	1 (2.7)	3 (8.1)	33 (89.1)
I accept egg donation to give my partner a genetic child	9 (24.3)	12 (32.4)	16 (43.2)
Women who receive donated eggs could have mixed feelings about their child	2 (5.41)	7 (18.9)	28 (75.6)
I chose egg reception instead of adoption because I have a strong desire of experiencing pregnancy	21 (56.7)	10 (27.0)	6 (16.2)
It is important that parents talk to their kids about how they were conceived	11 (29.7)	14 (37.8)	12 (32.4)
No one besides the parents and the medical team should know how the child was conceived	14 (37.8)	16 (43.2)	7 (18.9)
Comparisons of appearance between mother and son/daughter will not affect me	23 (62.1)	10 (27.0)	4 (10.8)
If people knew how my child was conceived, they would discriminate him/her	3 (8.1)	6 (16.2)	28 (75.6)
It is important that my family knows that my child was conceived by a donated egg	7 (18.9)	4 (10.8)	26 (70.2)
It is important that the pediatrician or doctor of my child knows that he/she was conceived by a donated egg	23 (62.1)	8 (21.6)	6 (16.2)
I feel inferior as a woman for recurring to egg reception in order to have a child	(0.0)	6 (16.2)	31 (83.7)
It is important for the child’s identity to know where he/she is from	6 (16.2)	11 (29.7)	20 (54.0)
If you are thinking of keeping the egg receiving a secret, you are doing it to protect the child	22 (59.4)	10 (27.0)	5 (13.5)
If my child found out that I have hidden his/her origins from him/her, it would damage our relationship	8 (21.6)	10 (27.0)	19 (51.3)
I feel capable of handling negative or embarrassing comments about using donated eggs	23 (62.1)	10 (27.0)	4 (10.8)

Number of patients = 37.

The responses of the patients reveal distinct emotional and psychological profiles related to receiving donated eggs, with some variations based on the acceptance of egg donation to conceive a genetic child, the difficulty of accepting the loss of their genetic load, and discomfort in having a child conceived with donor gametes. Across all statements, most patients expressed optimism and hope for the process, while negative emotions such as fear and distress were presented in smaller proportions. Despite variations found, these results did not present statistical differences when compared, as observed in Tables 4, 5 e 6.

**Table 4:** The emotional aspects regarding the following statement agreement analysis: *“I accept egg donation to give my partner a genetic child”.*

Corresponding answers number (%)	Agree/Partially agree	Disagree	*p*
	n=21	n=16	
Calm	14 (66)	8 (50)	0.336^*^
Frightened	9 (42.8)	8 (50)	
Optimistic	19 (90.4)	15 (93.7)	
Hopeful	21 (100)	16 (100)	
Anguished	5 (23.8)	4 (25)	
Have someone to share with	18 (85.7)	14 (87.5)	
Previous psychological or psychiatric treatment	12 (57)	10 (62.5)	
Current psychological or psychiatric treatment	4 (19)	7 (43.7)	

n: Number of patients

* Fisher’s exact test and chi-square test, considering *p*<0.05

**Table 5:** The emotional aspects regarding the following statement agreement analysis: *“It is difficult to accept the loss of my genetic load”.*

Corresponding answers number (%)	Agree/Partially agree	Disagree	*p*
	n=23	n=14	
Calm	12 (52)	10 (71.4)	0.314^*^
Frightened	13 (56.5)	4 (28.5)	
Optimistic	21 (91.3)	13 (92.8)	
Hopeful	23 (100)	14 (100)	
Anguished	7 (30.4)	2 (14.2)	
Have someone to share with	18 (78.2)	14 (100)	
Previous psychological or psychiatric treatment	16 (69.5)	6 (42.8)	
Current psychological or psychiatric treatment	8 (34.7)	3 (21.4)	

n: Number of patients

* Fisher’s exact test and chi-square test, considering *p*<0.05

**Table 6:** The emotional aspects regarding the following statement agreement analysis: *“The feelings that surround having a child conceived through gamete donation bothers me”.*

Corresponding answers number (%)	Agree/Partially agree	Disagree	*p*
	n=09	n=28	
Calm	4 (44)	18 (64.2)	0.438^*^
Frightened	6 (66)	11 (39.2)	
Optimistic	8 (88)	26 (92.8)	
Hopeful	9 (100)	28 (100)	
Anguished	4 (44)	5 (17.8)	
Have someone to share with	7 (77)	25 (89.2)	
Previous psychological or psychiatric treatment	7 (77)	15 (53.5)	
Current psychological or psychiatric treatment	3 (33)	8 (28.5)	

Number of patients = 39.

## DISCUSSION

The study evaluated the emotional impact and psychological aspects involved in the treatment with donor oocytes, looking at these emblematic issues related to receiving donated eggs. Based on our results, a substantial number of patients initially found it difficult or only accepted the treatment because they had no other option. On the other hand, a considerable number of patients agreed at least in part with the phrase “*It is difficult to accept the loss of my genetic load*” and with the phrase “*I accept egg donation to give my partner a genetic child*”. These results indicate that egg donation involves a process of acceptance, mourning for their genetic capital and the transformation of what they understood as the concept of motherhood, in line with previous data in the literature (Applegarth *et al*., 2016). Accepting the use of donated eggs is easier for some couples than others, but according to Greenfield D.A., the process very often involves some emotional loss: of a genetic link with the child, the continuation of the family line and, for many women, the fear of “not seeing themselves in the baby” (usually a fear of not having a normal bond with the baby) (Greenfeld, 2015).

Regarding the emotions reported in the questionnaire by the participants, anxiety, anguish and fear are frequent feelings in this process, even in those participants who were also calm. In addition, this data reflects the variety of concomitant emotions in this process, reinforcing the importance of maintaining a mental health monitoring in these situations. Paradoxically, most patients were not in psychological or psychiatric treatment at the time of the assessment. A recent Brazilian study which offered psychological counseling during treatment with donated eggs showed that only 54.5% of couples adhered to the sessions. Considering the emotional complexity involved in the treatment and future implications, the authors suggest a more emphatic recommendation and encouragement from the professionals involved in the treatment, emphasizing its objectives and benefits (Montagnini *et al*., 2023).

Optimism and hope were also frequent, likely due to the expectation of a higher probability of pregnancy from egg donation when compared to one’s own eggs, as already demonstrated in the literature (Savasi *et al*., 2016). Most patients answered “no” to the following sentence: “*no matter how much a woman loves her child born through egg donation, she will never feel that it is really hers*”; and to: “*the feelings about having a child conceived through the use of a donor gamete bother me*”. This suggests that the desire for pregnancy is a predominant feeling during this process.

As for having support, most patients reported having someone to share their feelings with. Montagnini *et al*. (2023) analyzed the emotional aspects associated with egg donation and stated that psycho-emotional acceptance varies significantly between women in a stable relationship and single women, concluding that the presence of emotional support seems to influence this experience and highlighting the importance of providing adequate psychological support, especially to those who do not have continuous emotional support in a stable relationship (Montagnini *et al*., 2023).

As for revealing how the child was conceived, this aspect was not considered important by one third of the participants in the study, but the answers indicate a significant increase in the intention to reveal the origins, which points that they perceive disclosure as being important for the child. Most participants, considered it important for their child’s pediatrician or doctor to know that conception was achieved through egg donation, making it clear that they were concerned about the medical aspects involved in not knowing the donor’s health history. Those who would keep the child’s origin as a secret, they would do it in a form of protection, reinforcing parental care from preconception.

The literature brings controversial results regarding the importance of parents to tell their child how they conceived them. A longitudinal study, including 73 families with babies born through egg donation, identified that most mothers (75.3%) intended to reveal to their children the origin through egg reception (Lysons *et al*., 2023). Clare Murray and Susan Golombok published a study of 17 families formed through egg reception in the UK and 47% of parents had no intention of telling their children about the way they were conceived and 29% intended to tell them in the future (Murray & Golombok, 2003).

In our study, almost half of participants agreed at least in part that their relationship with their child could be damaged by the omission of their origin. It may happen due to a loss of trust in the relationship with their parents, after the omission of part of their history. This is in line with the literature, which demonstrates the potential effect of family secrets as a cause of instability in these bonds (Silva *et al*., 2023). Parents are often faced with having to make decisions about sharing information with their children on a wide variety of topics, including sexuality, death and origins. The timing and the child’s ability to understand must be considered, as well as sensitivity when passing on information. When children realize that information is being withheld, they can become anxious and confused, losing their sense of confidence and even blaming themselves (Farinati & Lopes, 2015).

The issue of anonymity can affect both donors and recipients, as well as the children generated in this process. The absence of information about the identity of donors can cause uncertainty and anguish for those involved (Blake *et al*., 2014). On the other hand, revealing the identity of the donor can introduce challenges and complexities into family dynamics and interpersonal relationships (Montagnini *et al*., 2023). The evolution of these practices will continue to be shaped by advances in ethics, legislation, and research into the psychological impact of parenting on egg donation processes. An interesting fact is that 89.19% answered “*no*” to the phrase “Y*ou fear that the donor will regret* her decision *and start looking for the child*”, showing that the patients trust the treatment and are not afraid the donor will seek information regarding the offspring.

In examining the emotional responses associated with the statements “*I accept egg donation to give my partner a genetic child*,” “*It is hard to accept the loss of my genetic load*,” and “*Feelings about having a child conceived with donor gametes bother me*,” no statistically significant differences were found, though some noteworthy variations emerged. Patients who agreed or partially agreed with these statements displayed similar levels of calm, fear, optimism, hopefulness, and distress when compared to those who disagreed. There was a trend suggesting varied emotional and psychological profiles among patients receiving donated eggs; however, these were not statistically distinct.

Acceptance of egg donation for conceiving a genetically related child to the partner and the difficulty in accepting the loss of genetic load showed a trend toward more complex emotional responses. These findings suggest that even patients initially uncomfortable with egg donation may develop a positive attitude toward the success of assisted reproduction. Regarding the acceptance of genetic loss, women who agreed or partially agreed with this statement tended to experience higher levels of fear, while those who disagreed tended to report greater calmness. This suggests that genetic loss may be linked to emotional stability during the egg donation process, where detachment from genetic expectations could reduce anxiety. Thus, although not statistically significant, the relationship between acceptance of egg donation and emotional well-being may be clinically relevant in reproductive medicine practice. Additionally, distress levels were reported similarly across groups. It is important to note that, despite these variations in emotions, fewer than half of the patients were receiving current psychological or psychiatric treatment, underscoring the need for a more comprehensive mental health approach to assisted reproduction care.

Finally, it is important to mention two studies: one carried out with 65 families from assisted reproduction, including 22 families with a surrogate uterus, 17 families with egg reception and 26 donor semen, compared to 52 families formed without the aid of reproductive techniques. This study found that the associations between parenting and child adjustment did not differ between assisted and spontaneous reproduction families, suggesting that the absence of a biological link between children and their parents does not interfere with the development of positive mother-child relationships or psychological adjustment in adulthood (Golombok *et al*., 2023). The second study evaluated 5-year-old children born through egg donation from the child’s perspective and showed that they viewed their family relationships and themselves positively, and in a very similar way to children born through IVF using their parents’ own gametes (Imrie *et al*., 2022). Both studies showed reassuring outcomes for the parents that used donated eggs to create their families.

## CONCLUSION

The study shows that most patients, who were all in stable relationships, accepted egg donation as a good treatment option. Even so, for many the choice was difficult, suggesting that receiving donated eggs is associated with a process of acceptance and mourning of their genetic capital. Feelings such as anxiety and calmness were present in most of the patients, reflecting the variety of emotions concurrent with this process.

Differences in emotions regarding the acceptance of egg donation were not statistically different. Nevertheless, the variation shown in this study may be clinically relevant and should be considered. Patients who internalize the concept of donation without discomfort tended to exhibit more positive emotional responses, with high levels of optimism and hope. These results underscore the importance of exploring patients’ perceptions and providing targeted psychological support to enhance the treatment experience and improve emotional well-being during the use of donated eggs.

The dilemma of whether to tell their offspring about their genetic origin was present, as several previous studies have pointed out. Therefore, constant care and attention to emotional issues are essential for clinicians to guide these patients through all the questions involved in the egg donation treatment.

## References

[r1] Agência Nacional de Vigilância Sanitária -
ANVISA (2024). SisEmbrio- Relatório do Sistema Nacional de
Produção de Embriões.

[r2] Applegarth LD, Kaufman NL, Josephs-Sohan M, Christos PJ, Rosenwaks Z. (2016). Parental disclosure to offspring created with oocyte donation:
intentions versus reality. Hum Reprod.

[r3] Baird DT, Collins J, Egozcue J, Evers LH, Gianaroli L, Leridon H, Sunde A, Templeton A, Van Steirteghem A, Cohen J, Crosignani PG, Devroey P, Diedrich K, Fauser BC, Fraser L, Glasier A, Liebaers I, Mautone G, Penney G, Tarlatzis B, ESHRE Capri Workshop Group (2005). Fertility and ageing. Hum Reprod Update.

[r4] Blake L, Jadva V, Golombok S. (2014). Parent psychological adjustment, donor conception and disclosure:
a follow-up over 10 years. Hum Reprod.

[r5] Blakemore JK, Voigt P, Schiffman MR, Lee S, Besser AG, Fino ME. (2019). Experiences and psychological outcomes of the oocyte donor: a
survey of donors post-donation from one center. J Assist Reprod Genet.

[r6] Braga DP, Setti AS, Figueira RC, Azevedo Mde C, Iaconelli A (2016). Lo Turco EG, Borges E Jr. Freeze-all, oocyte vitrification, or
fresh embryo transfer? Lessons from an egg-sharing donation
program. Fertil Steril.

[r7] Colombo T, Hentschke MR, Badalotti M, Kira ATF, Telöken IB, Trindade VD, Dornelles VC, Petracco A, Wendland EM. (2023). A sharing oocyte donation program: a 15-year cohort
stud. JBRA Assist Reprod.

[r8] de Vet A, Laven JS, de Jong FH, Themmen AP, Fauser BC. (2002). Antimüllerian hormone serum levels: a putative marker for
ovarian aging. Fertil Steril.

[r9] Farinati DM, Lopes HP., Straube KM, Melamed RM (2015). Temas contemporâneos de Psicologia em reprodução
humana assistida - A infertilidade em seu espectro psicoemocional.

[r10] Ghelich-Khani S, Kazemi A, Fereidooni-Moghadam M, Alavi M. (2020). A mental health program for infertile couples undergoing oocyte
donation: protocol for a mixed methods study. Reprod Health.

[r11] Golombok S, Jones C, Hall P, Foley S, Imrie S, Jadva V. (2023). A longitudinal study of families formed through third-party
assisted reproduction: Mother-child relationships and child adjustment from
infancy to adulthood. Dev Psychol.

[r12] Greenfeld DA. (2015). Effects and outcomes of third-party reproduction:
parents. Fertil Steril.

[r13] Imrie S, Lysons J, Jadva V, Shaw K, Grimmel J, Golombok S. (2022). Parent-child relationship quality and child psychological
adjustment in families created using eggdonation: children’s perspectives at
age 5 years. Hum Reprod.

[r14] Lasheras G, Mestre-Bach G, Clua E, Rodríguez I, Farré- Sender B. (2020). Cross-Border Reproductive Care: Psychological Distress in A
Sample of Women Undergoing In Vitro Fertilization Treatment with and without
Oocyte Donation. Int J Fertil Steril.

[r15] Legendre G, Catala L, Morinière C, Lacoeuille C, Boussion F, Sentilhes L, Descamps P. (2014). Relationship between ovarian cysts and infertility: what surgery
and when?. Fertil Steril.

[r16] Lutjen P, Trounson A, Leeton J, Findlay J, Wood C, Renou P. (1984). The establishment and maintenance of pregnancy using in vitro
fertilization and embryo donation in a patient with primary ovarian
failure. Nature.

[r17] Lysons J, Imrie S, Jadva V, Golombok S. (2023). Families created via identity-release egg donation: disclosure
and an exploration of donor threat in early childhood. Reprod Biomed Online.

[r18] Montagnini HL, Kimati CT, Lorenzon AR, Bonetti TC, Serafini PC, Motta E, Domingues TS. (2023). Psycho-emotional acceptance in couple and single women who choose
to undergo IVF treatment with donor eggs. JBRA Assist Reprod.

[r19] Murray C, Golombok S. (2003). To tell or not to tell: the decisionmaking process of
egg-donation parents. Hum Fertil.

[r20] Roudinesco E. (2003). A família em desordem.

[r21] Savasi VM, Mandia L, Laoreti A, Cetin I. (2016). Maternal and fetal outcomes in oocyte donation
pregnancies. Hum Reprod Update.

